# Effectiveness of prophylactic doses of tranexamic acid in reducing hemorrhagic events in sleeve gastrectomy

**DOI:** 10.1007/s00423-022-02630-5

**Published:** 2022-08-03

**Authors:** Paweł Lech, Maciej Michalik, Kamil Waczyński, Karolina Osowiecka, Natalia Dowgiałło-Gornowicz

**Affiliations:** 1 Department of General, Minimally Invasive and Elderly Surgery, Collegium Medicum, University of Warmia and Mazury, Niepodległości 44 St, 10-045 Olsztyn, Poland; 2 Department of General, Colorectal and Oncologic Surgery, Collegium Medicum, Nicolaus Copernicus University in Torun, Ujejskiego 75 St, 85-168 Bydgoszcz, Poland; 3 Department of Psychology and Sociology of Health and Public Health, School of Public Health, University of Warmia and Mazury, Warszawska 30 St, 10-041 Olsztyn, Poland

**Keywords:** Tranexamic acid, TXA, Hemorrhagic events, Laparoscopic sleeve gastrectomy

## Abstract

**Purpose:**

Laparoscopic sleeve gastrectomy (LSG) is currently the most common bariatric surgery in the world. Although it appears to be a safe treatment for obesity, it is still at risk of complications. The latest literature shows that postoperative bleeding occurs in 2–4% of cases, and up to 3% of cases requires reoperation for hemostasis. The aim of the study is to assess the effect of tranexamic acid (TXA) on hemorrhagic events and the reoperation rate in patients undergoing LSG.

**Methods:**

The study was designed as a retrospective analysis of patients undergoing LSG. We investigate the patients 6 months before and 6 months after introducing the prophylaxis doses of TXA into our bariatric protocol (non-TXA group vs TXA group).

**Results:**

Three hundred fourteen patients underwent LSG in a high-volume center from 2016 to 2017. After introducing TXA, a statistically significant reduction in the incidence of hemorrhage during surgery was observed (22.3% vs 10.8%, *p* = 0.006). There was a statistically significant reduction in the need for the staple line oversewing (10.2% vs 1.9%, *p* = 0.002). The mean operating time and the mean length of hospital stay were significantly higher in the non-TXA group than TXA group (63.1 vs 53.7 min, *p* < 000.1; 2.3 vs 2.1, *p* = 0.02). In both groups of patients, no venous thromboembolism or other complications occurred within 6 months after the surgery.

**Conclusions:**

The prophylactic doses of TXA may be useful in reducing the hemorrhagic events during LSG. It may also shorten the length of hospital stay and the operating time.

## Introduction

Laparoscopic sleeve gastrectomy (LSG) is currently the most common bariatric surgery in the world [1]. Although it appears to be a safe treatment for obesity, it is still at risk of complications. The latest literature shows that postoperative bleeding occurs in 2–4% of cases, and up to 3% of cases requires reoperation for hemostasis [2–4].

By introducing to our daily practice enhanced recovery after bariatric surgery, we focus on the quick mobilization of the patient, shortening the length of hospital stay and discharge from the hospital on the first postoperative day [5]. Undoubtedly, it is beneficial both from the point of view of the patient and the economy of the facility. However, it gives less insight into the occurrence of possible complications, including hemorrhagic complications. Therefore, ways of reducing these risks are sought, such as the staple line oversewing, the use of reinforcement materials or fibrin sealants [6–8].

One of the effective methods of reducing the risk of postoperative bleeding is using tranexamic acid (TXA) [9]. It has been well described in cardiac or orthopedic surgery [10, 11]. TXA is an antifibrinolytic drug, a synthetic analog of lysine [12]. It prevents lysine binding sites on plasminogen by blocking plasmin formation and thus fibrinolysis. There are only a few reports of the use of TXA in postoperative bleeding following bariatric surgery [13–15].

The aim of the study is to assess the effect of TXA on hemorrhagic events and the reoperation rate in patients undergoing LSG.

## Materials and methods

The study was designed as a retrospective analysis of patients undergoing LSG. Patients were qualified for surgery according to national and international criteria. We investigate the patients before and after introducing the prophylaxis doses of TXA into our bariatric protocol (non-TXA group vs TXA group). To avoid potential bias due to the learning curve, experience, and time of treatment, the same team of surgeons performed the surgery. The surgeons’ experience is over 150 procedures a year. Additionally, we include patients operated 6 months before and after the introduction of TXA.

Following our bariatric protocol, 1 g of intravenous TXA (Exacyl ®) was routinely administered as an induction to all LSG patients and three times after the surgery every 8 h. As written in our national Summary of Product Characteristics. In parallel, as part of thrombolytic prophylaxis, 40 mg of low molecular weight heparin (Clexane ®) was administered subcutaneously 12 h before the surgery, 12 h after the surgery, and once a day for 10 days after the discharge from the hospital. Patients were discharged on the first postoperative day, when they were able to self-mobilize and consume adequate amounts of oral fluids. Otherwise, or in the event of complications, they stay in the hospital longer. Thirty days after surgery, each patient showed up for a personal, routine, post-surgery visit to the outpatient clinic. A physical examination and laboratory tests were performed. The subsequent visits in the 6th, 12th month, and annually after the surgery can take place during both personal visits and telephone consultations.

### Surgical technique

All LSGs were performed according to the standard procedure described by Bhandari et al. [16]. An ultrasonic dissection device (Medtronic, Covidien, Inc.) and a 36-F bougie size were used. The sleeve started 2–4 cm from pylorus. The Endo GIA™ Ultra Universal Staplers (Medtronic, Covidien, Inc.) were used with 60-mm cartridge depending on stomach thickness. Typically, 3 purple and 2 blue cartridge were used. No staple line reinforcement or buttressing was used. Before the end of surgery, the pneumoperitoneum was lowered to 10 mmHg to check for evidence of bleeding. The clips were placed in case of bleeding points of staple line. If the bleeding cannot be controlled by clipping, the surgeon decided to perform the staple line oversewing with 3–0 PDS stich. The intra-abdominal drainage was not used routinely.

### Outcomes measured

**Fig. 1:**
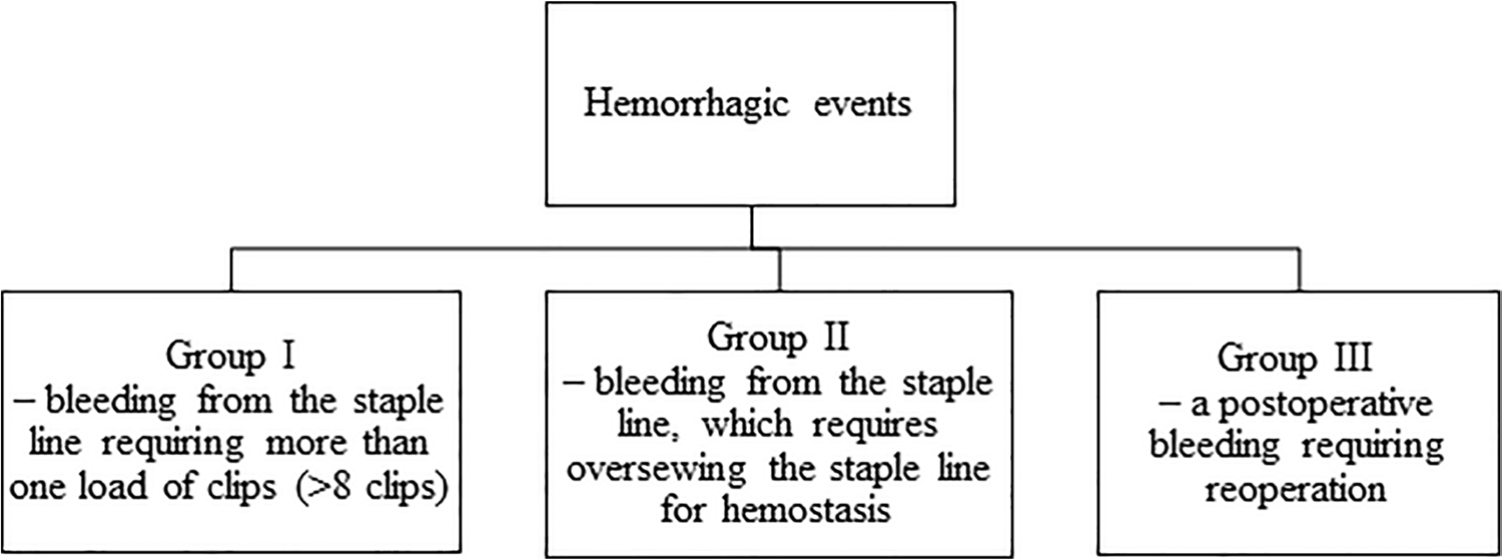
Definition of hemorrhagic events during and after the surgery

We analyzed the number of hemorrhagic events during and after the surgery. We divide it into 3 groups, Fig. 1. The first is bleeding from the staple line requiring more than one load of clips (> 8 clips). Second, there is bleeding from the staple line, which requires oversewing the staple line for hemostasis. Third is a postoperative bleeding requiring reoperation. Secondary outcomes included the pre- and postoperative levels of hemoglobin, hematocrit, and red blood cells; operating time, length of hospital stay, and the occurrence of 30-day complications, and venous thromboembolism within 6 months after surgery. Anemia 30 days after the surgery was defined as hemoglobin < 10 mg/dL.

### Statistical analysis

The data was calculated using descriptive statistics. The chi-square test was used to compare the proportion between non-TXA and TXA group due to various factors. The differences in mean age, body mass index, operating time, length of hospital stay, and laboratory parameters between non-TXA and TXA group were assessed using Student *t* test. A *p* value < 0.05 was considered significant. The analysis was conducted using Statistica (data analysis software system), version 13, http://statistica.io (accessed on 1 October 2021) TIBCO Software Inc., Krakow, Poland (2017).

## Results

Three hundred fourteen patients underwent LSG in a high-volume center from 2016 to 2017. Twenty-three patients were lost in follow-up at the 6th month. The follow-up rate was 92.7%. The first group of patients (non-TXA group) consisted of 124 women and 33 men (79%, 21%) and was operated without introducing the prophylactic doses of TXA. The second group (TXA group) had 124 women and 33 men (79%, 21%) and was operated using the prophylactic doses of TXA. The TXA group was statistically older than non-TXA group (*p* = 0.048), but there were no significant differences in body mass index, type 2 diabetes mellitus, and arterial hypertension (*p* > 0.05), Table 1.

**Table 1:** Characteristics of groups (results in bold indicate statistical significance,* p* < 0.05)

	Non-TXA group	%	TXA group	%	*p*
Sex					
Woman	124	79	124	79	
Man	33	21	33	21	
Mean age (± SD) (years)	39 (11.2)		41.6 (11.5)		**0.048**
Mean BMI (± SD) (kg/m^2^)	44.6 (6.6)		46.0 (9.6)		0.16
Diabetes mellitus type 2					
Yes	26	16.6	33	21	0.31
No	131	83.4	124	79	
Arterial hypertension					
Yes	67	42.7	73	46.5	0.5
No	90	57.3	84	53.5	
Mean operating time (± SD) (min)	63.1 (19.2)		53.7 (14.5)		** < 0.001**
Mean length of hospital stay (± SD) (days)	2.3 (0.9)		2.1 (0.4)		**0.02**
Hemorrhagic incidents					
Yes	35	22.3	17	10.8	**0.006**
No	122	77.7	140	89.2	
Bleeding					
Yes	16	10.2	14	8.9	0.7
No	141	89.8	143	91.1	
Oversewing					
Yes	16	10.2	3	1.9	**0.002**
No	141	89.8	154	98.1	
Reoperations					
Yes	3	1.9	0	0	0.08
No	154	98.1	157	100	
Anemia					
Yes	11	7.0	4	2.5	0.06
No	146	93.0	153	97.5	
Laboratory tests					
Mean RBC difference before and after surgery (± SD)	0.37 (0.3)		0.42 (0.3)		0.23
Mean Hb difference before and after surgery (± SD)	1.12 (1.0)		1.17 (0.9)		0.66
Mean Ht difference before and after surgery (± SD)	3.69 (3.8)		3.9 (3.6)		0.63

*SD* standard deviation, *BMI* body mass index, *RBC* red blood count, *Ht* hematocrit, *Hb* hemoglobin

After introducing TXA as a prophylaxis into standard management, a statistically significant reduction in the incidence of hemorrhage during surgery was observed (22.3% vs 10.8%, *p* = 0.006). There was a statistically significant reduction in the need for the staple line oversewing (10.2% vs 1.9%, *p* = 0.002). However, no significant differences in the use of clips and reoperation rate were observed. Nevertheless, the reoperation rate in the analyzed time decreased to 0, *p* = 0.08. Only one patient required a transfusion in the non-TXA group and none in the TXA group; this is of no clinical significance and not statistically significant. We found no changes in mean difference in hemoglobin, hematocrit, and red blood cell levels before and after the surgery (*p* > 0.05). The mean operating time and the mean length of hospital stay were significantly higher in the non-TXA group than TXA group (63.1 vs 53.7 min, *p* < 000.1; 2.3 vs 2.1, *p* = 0.02), Table 1.

In both groups of patients, no evident, clinically noticeable venous thromboembolism or other complications occurred within 6 months after the surgery. The incidence of anemia in the first 30 days after surgery was lower in TXA group (2.5% vs 7%, *p* = 0.06), but not statistically significant, Table 1.

## Discussion

Our study analyzed the effect of TXA on perioperative bleeding in patients undergoing LSG. There are many reports in the literature regarding the use of TXA in various fields of surgery. However, little is known about its effects on bariatric surgery. To our knowledge, this is the first report to compare such a large group of bariatric patients to provide objective evidence.

TXA is synthetic lysine analog that inhibits the activation of plasminogen to plasmin [12]. It is routinely used in orthopedic and cardiac surgery to reduce the perioperative bleeding [9, 10]. Although the efficacy of TXA has been confirmed in studies, current studies have focused on effective dose adjustment as well as careful evaluation of use in elderly patients [10, 17]. Recent reports have shown that TXA can be safely used in elderly patients without increasing the risk of thrombotic complications [17, 18].

In a recent randomized controlled trial by Oseni et al., a significant reduction in blood was observed in woman undergoing emergency caesarean section after a prophylactic dose of TXA [19]. This was noticeable in reducing both intraoperative blood loss and postoperative hemoglobin levels.

The first report of the use of TXA in the prophylaxis of LSG was described by Chakravatty et al. [14]. They compared 25 patients who received 1 g of TXA intravenously as induction with the control group. They noticed that the introduction of TXA significantly decreases intraoperative bleeding and time of surgery, which is consistent with our observations. Hussain et al. showed in a letter to the editor that they also observed that the use of TXA may reduce postoperative transfusion rates and reoperation [13].

Klassen et al. analyzed patients who developed hemorrhage after bariatric surgery [15]. Treatment failure was observed in 4 of 44 complicated patients who received TXA after surgery. The authors suggested that TXA appeared to be safe in reducing the reoperation rate for bleeding.

The reported reoperation rate for bleeding in bariatric surgery ranges from 0.5 to 3% [3, 4]. In our study, the reoperation rate was 1.9% and it decreased to 0% after TXA in the presented group, but it was not statistically significant. In addition, we also noted shortening the operating time and the length of hospital stay. In our bariatric protocol, each patient requires 4 TXA ampoules, which costs less than PLN 10 (EUR 2.1). In contrast, buttressing materials or fibrin sealants cost about PLN 1000 (EUR 210). The use of TXA may be useful in reducing the overall costs of hospitalization in bariatric units. That may have a positive impact on the hospital’s finances. Importantly, we did not observe any evident thromboembolic events until 6 months after surgery.

Our study has several limitations. It includes the subjective definition of hemorrhage events, retrospective, and non-randomized character. However, to minimize the risk of bias, we included patients operated within 6 months before and after the introduction of TXA into the bariatric protocol. The team of bariatric surgeons in our department consists of two experienced surgeons who have operated for over 5 years and perform over 150 operations a year. The surgeons were not blinded, which may have influenced the decision to use more clips or to perform the staple line oversewing. But the statistics on reoperation rate and postoperative hemoglobin levels were absolute. However, a randomized controlled trial with blind surgeons appears to be an ideal model to conduct the trial, as proposed by Leeman et al. [20].

## Conclusions

The prophylactic doses of TXA may be useful in reducing the hemorrhagic events during LSG. It may also shorten the length of hospital stay and the operating time. However, more randomized controlled trials are needed.
